# Similarities and differences in the functions of non-suicidal self-injury (NSSI) across gender non-conforming and cisgender young adults

**DOI:** 10.1016/j.jad.2024.08.224

**Published:** 2024-09-02

**Authors:** Nina M. Lutz, Samuel R. Chamberlain, Jon E. Grant, Christine Lochner, Paul O. Wilkinson, Tamsin J. Ford, Sharon A.S. Neufeld

**Affiliations:** a Department of Psychiatry, University of Cambridge, UK; b The Mental Health Foundation, London, UK; c Department of Psychiatry, Faculty of Medicine, University of Southampton, UK; Southern Health NHS Foundation Trust, Southampton, UK; d Department of Psychiatry and Behavioral Neuroscience, University of Chicago, USA; e SA MRC Unit on Risk and Resilience in Mental Disorders, Department of Psychiatry, University of Stellenbosch, South Africa; f Cambridgeshire and Peterborough NHS Foundation Trust, UK

**Keywords:** NSSI, Self-harm, Functions, Gender, Transgender, Non-binary

## Abstract

**Background:**

Non-suicidal self-injury (NSSI) can be motivated by a broad range of functions and many individuals report multiple reasons for self-injuring. Most NSSI research has involved predominantly female samples and few studies have examined gender similarities and differences in function endorsement.

**Methods:**

We characterise the prevalence and versatility of NSSI functions within a gender-diverse online sample of cisgender women (cis-women; *n* = 280), cisgender men (cis-men; *n* = 176), and transgender, non-binary, and other gender non-conforming young adults (TGNC; *n* = 80) age 18–30 (*M* = 23.73, *SD* = 3.55). The Ottawa Self-Injury Inventory (OSI-F) assessed 24 intrapersonal and social functions across nine domains: affect regulation, self-punishment, anti-dissociation, anti-suicide, sensation seeking, sexuality, interpersonal influence, and body image.

**Results:**

TGNC participants and cis-women were significantly more likely to report intrapersonally motivated NSSI and greater function versatility than cis-men. Low mood, emotional distress, suicidality, and trauma symptomology appeared to contribute to gender differences in function endorsement. Gender similarities also emerged; across groups, intrapersonal functions were substantially more common than social functions, and the most endorsed domains were affect regulation and self-punishment. No domains were gender specific.

**Limitations:**

The OSI-F was developed from majority female samples and may not adequately capture the experiences of other gender groups.

**Conclusions:**

Interventions which reduce distress and strengthen emotion regulation are likely to benefit individuals who self-injure regardless of gender. However, most individuals report multiple NSSI functions and person-centred interventions which address this complexity are needed. Future research should develop gender-informed treatment models which consider the unique experiences of TGNC individuals and cis-men who self-injure.

## 1 Introduction

Non-suicidal self-injury (NSSI) is the deliberate, self-directed damage of body tissue without suicidal intent, often by cutting, burning, scratching, or hitting oneself (Klonsky et al., 2014). NSSI is significantly more common among women than men (Bresin and Schoenleber, 2015; Xiao et al., 2022), resulting in a field of research focused predominantly on the experiences of women (Millard, 2013). There is growing evidence that transgender, non-binary, and other gender non-conforming (TGNC) individuals are the gender identity group at highest risk of NSSI (Surace et al., 2021), however this population is absent from prior literature on gender differences in NSSI. Thus, there is a pressing need for research which investigates gendered gaps while considering gender identity in our understanding of NSSI engagement. The study of gender group similarities and differences in the prevalence and versatility of NSSI functions is one area requiring attention.

The emotion regulation properties of NSSI have received particular attention in the literature (Hooley and Franklin, 2018; McKenzie and Gross, 2014). Reviews of both qualitative and quantitative research conclude individuals most often self-injure to cope with negative feelings (Edmondson et al., 2016; Taylor et al., 2018). However, NSSI is a complex behaviour which can be motivated by a range of functions. These motives can be categorized as intrapersonal or social (Klonsky et al., 2015). Intrapersonal functions aim to directly change how one is feeling, including alleviating distress, tension, dissociation, or suicidal thoughts. Social functions aim to change one's environment by influencing how others feel or behave, including encouraging emotional support, ameliorating distressing situations (e.g., deterring abuse), or fitting in with peers (Edmondson et al., 2016; Klonsky, 2007). A 2018 meta-analysis estimates 74 % of people who self-injure report intra-personal functions and 44 % report social functions which are most often aimed at communicating distress (Taylor et al., 2018).

There have been no reviews or meta-analyses of gender differences in NSSI function endorsement, and nearly all prior studies had majority female samples which may obscure meaningful patterns of gender differences (Taylor et al., 2018). No studies have characterised NSSI functions within TGNC or LGBTQIA+ (lesbian, gay, bisexual, trans-gender, queer, intersex, asexual) samples or compared them with cis-gender individuals. However, the limited existing literature has consistently reported that intrapersonal functions are more strongly endorsed by women than men. This pattern is evident in community samples of adolescents (Laye-Gindhu and Schonert-Reichl, 2005; Zetterqvist et al., 2013), university students (Whitlock et al., 2011), and both adolescent and adult psychiatric patients (Claes et al., 2007; Victor et al., 2018, 2016). Thus, it appears a larger proportion of women who self-injure do so to regulate negative internal emotional states, compared to men who self-injure.

Conversely, the literature on gender differences in social functions of NSSI is conflicting. Some literature indicates men engage in more socially motivated NSSI than women. Early studies of gender differences found greater endorsement of social functions among adolescent boys (Laye-Gindhu and Schonert-Reichl, 2005) and higher rates of self-injuring in social settings among male college students (Whitlock et al., 2011) compared to female participants. This led some researchers to theorize gender differences in the prominence of social versus intra-personal reinforcement pathways which could translate to different mechanisms for clinical intervention (Green and Jakupcak, 2015). However, there is little empirical support for this purported difference. Though the lack of meta-analyses makes it difficult to draw conclusions, several studies report no gender differences in social function endorsement (Claes et al., 2007; Victor et al., 2018, 2016; Zetterqvist et al., 2013). These studies also find that both men and women consistently endorse intrapersonal functions more strongly than social functions, indicating NSSI is primarily intra-personally motivated across genders – however, this has yet to be investigated among TGNC individuals.

Moreover, many individuals report multiple reasons for self-injuring, though this function versatility has received little research attention. Coppersmith et al. (2021) were among the first to examine variability in NSSI functions over time and within episodes of NSSI. Using Ecological Momentary Assessment to collect real-time data from both clinical and community-based adolescent and adult samples, the authors found most participants (84 %) reported different functions across NSSI episodes and at least a quarter reported multiple functions within a single episode (Coppersmith et al., 2021). Though samples were small and predominantly female (*n* = 31, 87 % female), findings concur with longitudinal evidence that 42 % of adolescents who self-injure report two or more functions per most recent episode at age 16 (*n* = 528, 80 % female), which increases to 75 % at age 21 (Gardner et al., 2021). Many individuals report both intrapersonal and social NSSI functions, though intrapersonal functions are much more common (Case et al., 2020; Dixon-Gordon et al., 2022; Gardner et al., 2021). Notably, endorsing multiple functions is associated with greater likelihood of continuing NSSI into young adulthood (Gardner et al., 2021) and more clinically severe NSSI (Case et al., 2020; Dixon-Gordon et al., 2022). No studies have reported on gender differences in NSSI function versatility, highlighting the need for further research.

### 1.1 The present study

To address these gaps in the literature, the present exploratory study characterised the similarities and differences in NSSI function endorsement between TGNC individuals, cisgender women, and cisgender men in an online sample of young adults (age 18–30). We focused on functions reported as ‘often’ or ‘always’ a reason for NSSI since these dominant motives likely have the greatest utility for clinical intervention. Comparisons considered group differences in the prevalence and versatility of functions, presented within “intrapersonal” and “social” categories. Age and duration of NSSI history were tested as covariates based on longitudinal findings indicating an accumulation of NSSI functions in young people over time (Gardner et al., 2021).

From the existing literature on gender differences in NSSI functions, we hypothesized: (1) a larger proportion of cisgender women would report intrapersonal functions compared to cisgender men, and (2) intrapersonal functions would be more prevalent than social functions among both cisgender women and cisgender men. There is little evidence to support other a priori hypothesized similarities or differences, particularly pertaining to TGNC participants who have not previously been included in research characterising NSSI functions. Exploratory analyses therefore tested group differences across nine domains: affect regulation, self-punishment, anti-dissociation, anti-suicide, sensation seeking, sexuality, interpersonal influence, and body image. Comparisons of function versatility remained exploratory due to the lack of extant literature on gender differences in this NSSI characteristic.

## 2 Methods

### 2.1 Sample

Participants were recruited between May–December 2021 to complete an online mental health survey via Prolific.com, a global UK-based online research platform. Inclusion criteria were: age 18–30, residing in the United Kingdom or Unites States of America, and able to undertake the study procedures with internet access. There were no exclusion criteria. All eligible Prolific users were invited via email to participate. The invitation stated that participants would be asked about experiences of self-harm. Demographic pre-screeners were set to recruit a minimum of 100 TGNC participants to ensure sufficient group size for statistical analysis.

An initial 1763 survey responses were recorded, of which 233 duplicates were removed, 16 were rejected for failing attention checks, six exited before the NSSI items, three endorsed lifetime NSSI without responding to subsequent questions about NSSI history, and one did not report their gender. The full sample therefore included 1504 young adults (*n* = 103 TGNC), of whom 570 (37.9 %) reported NSSI history and responded to the OSI-F regarding NSSI functions at any point in their life. A further 24 participants were excluded who endorsed all 24 OSI-F items, an unlikely outcome which may reflect inattentive or dishonest responding. Thus, 546 individuals were included in the present study. The study was reviewed and approved by the Cambridge Psychology Research Ethics Committee (reference: PRE.2020.141, Principal Investigator Prof Sam Chamberlain). Participants provided electronic informed consent and were compensated up to £14/US$17 for their time.

### 2.2 Measures

#### 2.2.1 Gender identity

Participants responded to two questions: “What is your gender identity? Female, Male, Non-binary, or Other (write-in)” and “Do you identify as transgender? Yes/No”. Participants who identified as female or male and not transgender were respectively categorized as cisgender women and men. Participants who identified as transgender, non-binary, or another gender identity were categorized as TGNC.

#### 2.2.2 NSSI history

Lifetime NSSI history was assessed with a yes/no question from the validated Drugs, Alcohol and Self-Injury Questionnaire (DASI; Wilkinson et al., 2018): “Have you ever tried to hurt yourself on purpose without trying to kill yourself (for example: things like burning, cutting, or scratching yourself)?”. Participants who endorsed lifetime NSSI were asked follow-up questions including whether they had self-injured within the past year and how old they were the first time they self-injured, used here to calculate duration of NSSI history for the purpose of function versatility sensitivity analyses.

#### 2.2.3 Ottawa Self-Injury Inventory Functions scale (OSI-F)

The Ottawa Self-Injury Inventory Functions scale (OSI-F v.3.1; Nixon and Cloutier, 2005) lists 24 NSSI functions and asks participants to rate how often each function was a reason for starting or continuing to self-injure, on a 5-point Likert scale from “never a reason” to “always a reason”. Binary variables for each item, domain, and category were created, indicating whether the participant endorsed that item (or an item within that category/domain) as ‘often’ or ‘always’ a reason for self-injuring, allowing our interpretation to focus on participants' dominant NSSI motives.

Results are presented within the categories *Intrapersonal* and *Social* and described using domains characterised by Klonsky and colleagues (2007; 2015). Since the OSI-F was informed by Klonsky (2007), most items could be easily categorized. Our primary analyses did not use the proposed OSI-F four-factor structure as it has not been validated across gender groups. However, we referred to the OSI-F four-factor structure (Martin et al., 2013; Nixon et al., 2015) in cases of item categorization ambiguity. No statistical analyses were carried out to inform function grouping.

For the sake of clarity, the *Intrapersonal* domain *sexuality* was added to capture two items pertaining to sexual arousal/excitement which were not a subject of Klonsky’s research (2007, 2015) and load onto separate OSI-F factors (with low factor loadings) in the four-factor model (Martin et al., 2013; Guéerin-Marion et al., 2018). Additionally, the *Social* domain *body image* was added to categorise the single item “to change my body image and/or appearance” which loads onto the Social Influence factor of the OSI-F (Martin et al., 2013; Nixon et al., 2015), but was absent from Klonsky’s models (2007, 2015) and does not conceptually fit into the other domains presented here.

### 2.3 Statistical analysis

Analyses were conducted with STATA 14.2, significance threshold *p* < 0.05. Due to the exploratory nature of the analyses, we did not correct for multiple comparisons. A one-way ANOVA followed by Tukey post-hoc tests compared the average age of each gender group. Binary logistic regressions tested associations between age and each function category, domain, and item. Associations between age and function versatility were tested by linear regressions with robust estimators, including 3-level gender as a covariate. If associations were significant, age would be included as a covariate in the respective gender group comparisons.

Binary logistic regressions followed by the “lincom” function tested pairwise gender group differences in the prevalence of each function category, domain, and individual OSI-F item, including age as a covariate if indicated. Gender groups were compared on two continuous measures of function versatility: number of function domains and number of individual items endorsed as often or always a reason for NSSI. Gender group differences on these two measures were tested by linear regressions followed by “lincom” for pairwise comparisons, including robust estimators and age as a covariate. Effect sizes were calculated as Cohen's *d*.

Groups were also compared on the proportion of participants endorsing only intrapersonal functions, only social functions, both categories, or neither category as often or always a reason for NSSI. Dummy variables were generated for each of these four possibilities, and gender group differences were tested by binary logistic regressions followed by “lincom” for pairwise comparisons.

Within the sub-sample of participants reporting past-year NSSI, sensitivity analyses considered whether gender differences in duration of NSSI history confounded gender differences in function versatility. Duration of NSSI history (in years) was approximated by subtracting reported NSSI age of onset from current age. Duration could not be calculated for those without past-year NSSI because participants did not report when they last self-injured. A one-way ANOVA compared average NSSI duration across gender groups. If groups significantly differed, linear regressions (total number of items and domains) and binary logistic regressions (endorsement of intrapersonal and/or social functions) would be repeated in the past-year NSSI subsample including covariate NSSI duration.

## 3 Results

### 3.1 Participants

Participants were 546 young adults aged 18–30 years (*M* = 23.73, *SD* = 3.55) with histories of NSSI. This included 280 cisgender women (“cis-women”), 176 cisgender men (“cis-men”), and 80 TGNC participants. TGNC participants identified as non-binary (*n* = 54), transgender men (*n* =15), transgender women (*n* < 10), genderfluid (*n* < 10), genderqueer (*n* < 10), and bi-gender (*n* < 10). Participants resided in the United Kingdom (52.75 %) or United States of America (47.25 %) and described their ethnicity as white (72.89 %); Asian, South Asian, or Southeast Asian (8.24 %); Hispanic or Latino (7.88 %); Black African or Caribbean (4.21 %); multiple ethnicities (5.31 %); Middle Eastern (0.55 %); Native or Indigenous group (0.37 %); or other ethnic group (0.55 %).

### 3.2 Age effects

Gender groups significantly differed in age (F(2, 543) = 7.13, *p* < 0.001); cis-men (*M* = 24.47, *SD* = 3.68) were significantly older than both cis-women (*M* = 23.54, *SD* = 3.54, *p* = 0.016) and TGNC participants (*M* = 22.3, *SD* = 2.97, *p* = 0.001), though cis-women and TGNC participants did not differ (*p* = 0.22). Many of the function categories, domains, and items were significantly associated with age (Table 1, Table 2) and age was therefore included as a covariate in group comparisons of function endorsement.

Including three-level gender as a covariate, older age was significantly associated with endorsement of fewer function domains (*b* = −0.07, 95 % CI [−0.11,−0.03], *p* 0.001) and fewer items (*b* = −0.14, 95 % CI [−0.24,−0.05], *p* = 0.004) as often or always a reason for NSSI. Age was therefore included as a covariate in group comparisons of function versatility.

### 3.3 Intrapersonal functions

**Fig. 1:**
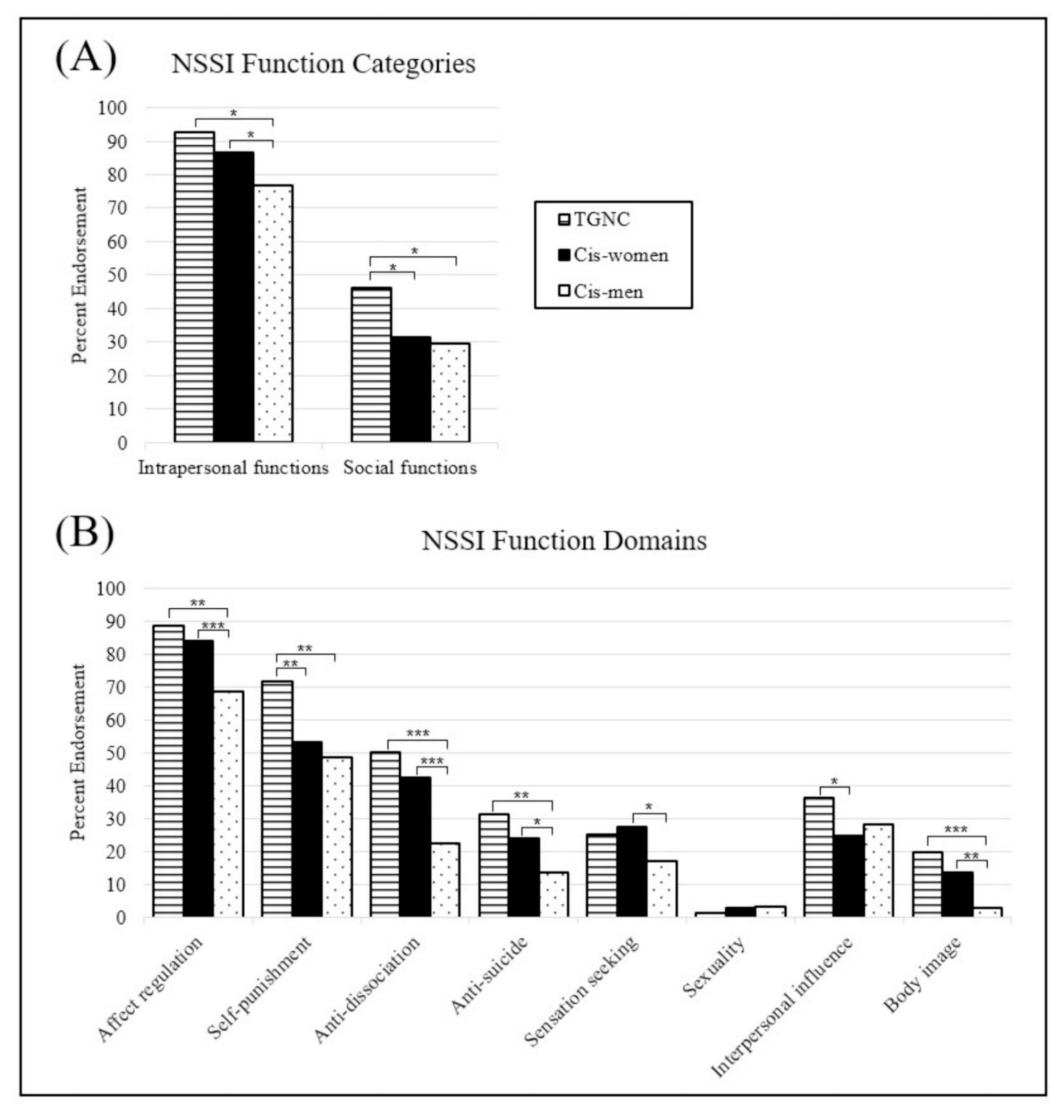
Proportions of transgender and other gender non-conforming (TGNC) participants, cisgender women (Cis-women), and cisgender men (Cis-men) who reported each function category (*A*) and function domain (*B*) as often or always a reason for their NSSI. Statistical comparisons are presented in Table 1. (*** indicates *p* < 0.01, ** indicates *p* < 0.01, * indicates *p* < 0.05).

Most participants with NSSI in each gender group reported at least one intrapersonal function as often or always a reason for NSSI (92.50 % TGNC, 86.55 % cis-women, 76.70 % cis-men). A significantly higher proportion of TGNC participants (*p* = 0.004) and cis-women (*p* = 0.007) reported intrapersonal functions as often or always a reason for NSSI compared to cis-men, though TGNC participants and cis-women did not significantly differ (Table 1, Fig. 1).

TGNC participants were significantly more likely than cis-men to endorse the intrapersonal domains of affection regulation (*p*= 0.001), self-punishment (*p* = 0.001), anti-dissociation (*p* < 0.001), and anti-suicide (*p* = 0.001) as often or always a reason for NSSI (Table 1, Table 2, Fig. 1). TGNC participants were also significantly more likely than cis-women to endorse self-punishment as often or always a reason for NSSI (*p* = 0.003). Cis-women were significantly more likely than cis-men to endorse affect regulation (*p* < 0.001), anti-dissociation (*p* < 0.001), anti-suicide (*p* = 0.007), and sensation seeking (*p* = 0.010) as often or always a reason for NSSI. Endorsement of the sexuality domain was notably low (below 4 % per group) and did not significantly differ across groups.

### 3.4 Social functions

At least one social function was endorsed as often or always a reason for NSSI by 46.25 % of TGNC participants, 31.38 % of cis-women, and 29.55 % of cis-men (Table 1, Fig. 1). TGNC participants were significantly more likely to endorse social functions as often or always a reason for NSSI compared to cis-men (*p* = 0.010) or cis-women (*p* = 0.014). Cis-women and cis-men did not significantly differ in this regard.

TGNC participants were significantly more likely than cis-women (*p* = 0.044) to endorse the interpersonal influence domain as often or always a reason for NSSI, however, these groups did not significantly differ on any individual function (Table 1, Table 2, Fig. 1). Cis-men did not significantly differ from cis-women or TGNC participants on overall endorsement of this domain or any individual function. Both TGNC participants (*p* < 0.001) and cis-women (*p* = 0.001) were significantly more likely than cis-men to endorse the single item in the body image domain (“to change my body image or appearance”) as often or always a reason for NSSI (Table 1, Table 2, Fig. 1). Cis-women and TGNC participants did not significantly differ in this regard.

### 3.5 Versatility of functions

#### 3.5.1 Gender group comparisons

Multiple functions (two or more) were endorsed as often or always a reason for NSSI by 90 % of TGNC participants, 81.72 % of cis-women, and 69.32 % of cis-men. Cis-men reported significantly less function versatility than cis-women or TGNC participants, evident in both the number of function domains and total number of items endorsed (*p*'s < 0.001; Table 3). Cis-women and TGNC participants did not significantly differ in the number of domains endorsed as often or always a reason for NSSI, though the total number of items was slightly but significantly higher among TGNC participants (*p* = 0.035; Table 3).

**Fig. 2:**
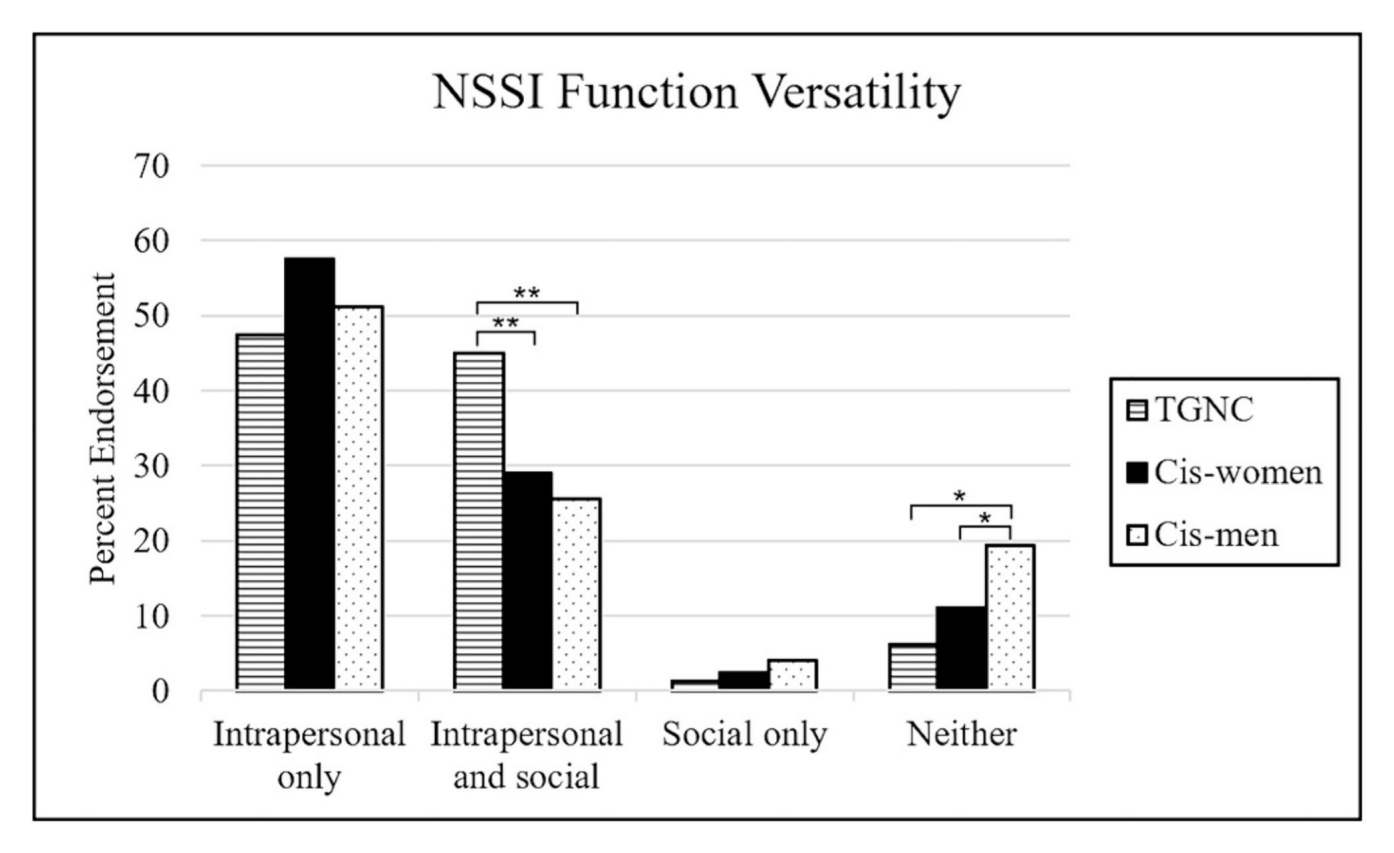
Proportions of transgender and other gender non-conforming (TGNC) participants, cisgender women (Cis-women), and cisgender men (Cis-men) who reported intrapersonal and/or social functions as often or always a reason for their NSSI. Statistical comparisons are presented in Table 4. (** indicates *p* < 0.01, * indicates *p* < 0.05).

In parallel with endorsing a greater number of functions as often or always a reason for NSSI, TGNC participants were significantly more likely than cis-men (*p* = 0.002) or cis-women (*p* = 0.007) to endorse both intrapersonal and social functions (Table 4, Fig. 2). Groups did not significantly differ in the proportion of participants reporting only intrapersonal or only social functions. Cis-men were significantly more likely than cis-women (*p* = 0.014) or TGNC participants (*p* = 0.011) to endorse none of the included functions as often or always a reason for NSSI (Table 4, Fig. 2). For all groups, intrapersonal functions were substantially more common than social functions.

#### 3.5.2 Sensitivity analysis – duration of NSSI history

Within the sub-sample of 248 participants endorsing past-year NSSI (47 TGNC, 127 cis-women, 74 cis-men), reported NSSI age of onset was subtracted from current age to approximate duration of NSSI history (range 0–21 years, *M* = 8.94, *SD* = 4.68). Gender groups did not significantly differ in duration of NSSI history (F(2, 245) = 0.50, *p* = 0.61). Therefore, versatility analyses were not repeated with duration of NSSI history as a covariate.

## 4 Discussion

The present study characterises the prevalence and versatility of NSSI functions endorsed by TGNC young adults, cis-women, and cis-men with histories of NSSI. This redresses limited and conflicting prior findings on NSSI functions in cis-men versus cis-women and a lack of work assessing gender differences in NSSI function versatility. Furthermore, to our knowledge this is the first study characterising NSSI functions in a sample of GNC individuals. Results supported both a priori hypotheses, revealing notable gender similarities and differences with implications for future gender-informed NSSI research and intervention development.

As hypothesized, a significantly higher proportion of cis-women endorsed intrapersonal functions as often or always a reason for NSSI compared to cis-men. These functions were based on seven domains of affect regulation, self-punishment, anti-dissociation, anti-suicide, sensation seeking, and sexuality. TGNC participants were also significantly more likely than cis-men to endorse intrapersonal functions as often or always a reason for NSSI, though they did not significantly differ from cis-women. Compared to cis-men, both TGNC individuals and cis-women were significantly more likely to report functions conceptually related to depression including coping with feeling sad, down, alone, and empty, though TGNC individuals and cis-women did not significantly differ from each other on these functions. Within the affect regulation domain, group differences in self-injuring to cope with un-bearable emotional pain showed the largest effect sizes, illustrating the mental anguish which often motivates NSSI especially for TGNC individuals and cis-women. Findings therefore align with previous studies reporting significantly higher intrapersonal function endorsement among women than men (Victor et al., 2018; Zetterqvist et al., 2013). Results also mirror prior analyses in the present sample and other adolescent and young adults samples showing that significantly elevated levels of psychological distress (i.e., depression and anxiety symptomology) and emotion dysregulation statistically mediate gender disparities in rates of NSSI (Lutz, 2022; Lutz et al., 2023; Wilkinson et al., 2022). Thus, it appears TGNC individuals and cis-women are more likely to experience high levels of emotional distress than cis-men, which translates to a larger proportion engaging in intrapersonally-motivated NSSI to cope with these negative feelings.

Results also revealed core similarities between groups. Consistent with our second hypothesis, within each gender group intrapersonal functions were substantially more common than social functions, encompassing interpersonal influence and body image domains. Affect regulation was the most endorsed domain by each group, and within this domain, releasing tension was the most endorsed item. Self-punishment was the second highest endorsed domain by each group. No functions were gender specific – all domains were endorsed by participants from each group. Endorsement of social functions in the absence of intra-personal functions was notably low (below 4 % per group), consistent with recent findings from another young adult sample (Gardner et al., 2021). The least common functions were similar across groups, including self-injuring to increase or decrease sexual feelings, belong to a group, get out of doing something, or avoid getting into trouble (below 5 % per group).

These findings align with past reviews and meta-analyses of majority female samples indicating NSSI is primarily a means of affect regulation, tension reduction, and self-punishment across gender groups (Edmondson et al., 2016; Taylor et al., 2018), providing some of the first empirical support for the extension of these findings to TGNC individuals and cis-men who self-injure. This is an essential point of clarification with ramifications for our understanding of NSSI treatment. Findings here provide preliminary support for the generalisability across gender groups of prominent affect-regulation models of NSSI (e.g., Hooley and Franklin, 2018; Selby et al., 2008) and leading NSSI interventions which improve distress tolerance and emotion regulation, including Dialectical Behaviour Therapy (DBT). Though clinical research has primarily studied NSSI treatment in majority female samples (Kothgassner et al., 2020), recent studies with men and TGNC patients find positive effects of DBT (Anestis et al., 2020; Birt et al., 2022; Camp et al., 2024). However, most participants in the present study reported multiple NSSI functions, illustrating the variability both between and within individuals in their reasons for self-injuring and the need for personalised treatment models (Lewis and Hasking, 2021; Walsh, 2012).

Several key group differences indicate that further gender-informed research on NSSI functions is needed. Both TGNC participants and cis-women reported significantly greater function versatility than cis-men, reflected by a higher number of individual functions and greater diversity in function domains. Compared to both cis-men and cis-women, TGNC participants were significantly more likely to endorse both intrapersonal and social functions and reported a significantly higher number of individual functions. Thus, across versatility indicators, TGNC participants showed the greatest versatility and cis-men showed the least. In combination with recent research linking greater function versatility with clinical severity (Case et al., 2020; Dixon-Gordon et al., 2022), this suggests possible gender disparities in NSSI severity which requires future investigation. It also indicates that TGNC individuals who self-injure may be more likely than cisgender peers to require complex treatment which addresses multiple intrapersonal and social NSSI motives.

Items related to coping with trauma symptomology demonstrated notable differences across groups. TGNC participants and cis-women each had significantly higher endorsement of self-injuring to stop feeling numb or unreal, distract from unpleasant memories, and punish themselves compared to cis-men. Endorsement of self-punishment and distraction from unpleasant memories were also significantly higher among TGNC participants than cis-women. This mirrors evidence that TGNC individuals experience significantly more trauma than their cisgender peers (Biedermann et al., 2021). Self-injuring to escape dissociation and cope with intrusive memoires are specifically associated with trauma symptomology (Horowitz and Stermac, 2018; Smith et al., 2014), and self-punishment is conceptualised as a re-enactment of abuse and expression of trauma-related shame (Smith et al., 2014). Trauma is central to literature on NSSI aetiology (Liu et al., 2018; van der Kolk et al., 1991) and trauma exposure, particularly gender-based violence, is prominent in explanations for gender disparities in NSSI prevalence for both cis-women and TGNC individuals (Diamond, 2013; Hyman, 1999; Smith et al., 1998). Results therefore illustrate the importance of evaluating traumatic experiences and post-traumatic symptoms when investigating gender differences in NSSI engagement.

TGNC participants and cis-women were both significantly more likely than cis-men to report self-injuring to change their body image or appearance, but did not significantly differ from each other in this regard. This function produced the largest effect sizes of any domain, illustrating the relevance of body-related experiences to gender differences in NSSI. Women report more body dissatisfaction than men (Quittkat et al., 2019) and negative body image longitudinally mediates NSSI engagement among young women (Black et al., 2019). Body dissatisfaction and gender dysphoria contribute to NSSI among TGNC individuals (Mirabella et al., 2020; Morris and Galupo, 2019). However, functions related to body image and gender dysphoria are absent from prominent NSSI frameworks. Further research is needed to elaborate on this topic and explore intents behind the body modification, which may include both intrapersonal and social motives.

Both cis-women and TGNC participants were significantly more likely than cis-men to report anti-suicide functions. This parallels gender differences in suicidal ideation and attempts, which are more prevalent among women than men (Miranda-Mendizabal et al., 2019) and among TGNC individuals than cisgender peers (Surace et al., 2021). The prevalence of anti-suicide functions illustrates the potential danger of interventions that emphasise NSSI cessation without addressing underlying sources of distress and supporting alternative coping strategies. This applies particularly to TGNC individuals, who had the highest endorsement of anti-suicide functions here and are at high risk of death by suicide as a population (Ream, 2022). Cis-men are also more likely to die by suicide than cis-women (Alothman and Fogarty, 2020). Further research into gendered relationships between suicidality and NSSI is therefore needed.

Cis-women were significantly more likely than cis-men to report sensation seeking functions related to experiencing a “high” and “proving how much I can take”. This was unexpected since previous findings suggest young men are more likely to endorse sensation seeking functions than young women (Whitlock et al., 2011). Moreover, prior analyses of the present sample found cis-men reported significantly higher levels of sensation seeking impulsivity than cis-women and TGNC participants (Lutz, 2022), consistent with past meta-analytic findings of significantly elevated sensation seeking among men (Hyde, 2014). Findings here therefore indicate further gender-informed investigation is needed into sensation seeking NSSI functions.

Results revealed gaps in our understanding of NSSI among cis-men. Cis-men showed the lowest endorsement across most items and were significantly more likely than cis-women and TGNC participants to endorse none of the included functions as often or always a reason for their NSSI. This could suggest the dominant reasons why cis-men self-injure are not represented in the OSI-F, which is entirely possible since the questionnaire was developed from predominantly female samples. This gap in understanding reflects a long-standing knowledge deficit whereby NSSI among cis-men has likely been underreported due to NSSI assessments leaving out methods more common among cis-men, such as punching walls (Kimbrel et al., 2017). It may also indicate a larger proportion of cis-men are unable to articulate their reasons for self-injuring. Up to 20 % of participants in majority female samples do not know why they self-injure (Edmondson et al., 2016). Men may exhibit less awareness of their emotions than women (Nolen-Hoeksema, 2012), which could be reflected in a higher proportion self-injuring without a clear motive. Though the reasons are uncertain, generally low function endorsement by cis-men in this sample indicates foundational research is needed to develop our understanding of male NSSI and determine whether current assessments are suitable across gender groups.

### 4.1 Limitations

As participants were recruited via an online research platform to complete a paid survey which would include questions about NSSI, this convenience sample is not reflective of all young people engaging in NSSI and findings may not generalise to other settings or populations. Although the OSI-F is especially comprehensive, it is not an exhaustive list of functions and is unlikely to fully capture all participants' experiences of NSSI. In addition, the OSI-F was developed with adolescents and not all items generalised to adults (e.g., “stop my parents being angry with me”). The questionnaire and preceding conceptual literature were developed from majority female samples, meaning the experiences of cis-men and TGNC individuals are underrepresented. Our cross-sectional investigation relies on retrospective recall which may affect accuracy. Participants reported NSSI functions from any point in their life, and there may be differences in endorsement between individuals with current versus past NSSI which we could not evaluate as participants did not report on NSSI cessation. Social stigma (experienced or feared) attached to interpersonally motivated NSSI may result in lower endorsement of social functions.

### 4.2 Future directions

For a more accurate study of NSSI functions, future research should extend existing work using Ecological Momentary Assessment (EMA) for real-time data collection. This methodology may yield more clinically useful information about the causes and consequences of NSSI and opportunities for intervention (Koenig et al., 2021). For instance, versatility results in the present study do not clarify whether cis-women and TGNC participants are more likely to report multiple simultaneous reasons for NSSI and/or more frequent NSSI for a variety of different reasons. EMA data would also be less impacted by recall bias inherent to retrospective questionnaires. Current EMA work on NSSI functions is based on small, predominantly female samples; thus, studies on daily life experiences of cis-men and TGNC individuals who self-injure are especially needed.

The effects of age and duration of NSSI history on NSSI functions is another area for future research. In contrast with prior research (Gardner et al., 2021), older age was associated with less function versatility here, though our cross-sectional design precludes further investigation into age-related trends. Gender differences in NSSI functions and overall prevalence of different functions may vary across the lifespan. For instance, intrapersonal functions are more strongly reinforcing and predictive of future NSSI (Gardner et al., 2021; Hooley and Franklin, 2018), suggesting social function endorsement may be lower in samples with longer histories of NSSI. Longitudinal research is needed to evaluate developmental trajectories of NSSI and investigate changes in functions over time, as this could illuminate key points for intervention.

## 5 Conclusions

This study is the first to characterise NSSI functions in a sample of TGNC and cisgender participants, offering valuable insights into similarities and differences across gender groups. The scarcity of prior research on experiences of TGNC individuals and cis-men who self-injure undermines clinical understanding and may translate to poor provision of care. By developing gender-informed knowledge, we can improve treatment frameworks and clinical training, resulting in better support for people of all genders who self-injure.

Findings here illustrate TGNC young adults and cis-women who self-injure are more likely to report intrapersonally motivated NSSI and greater function versatility than cis-men. The specific intrapersonal functions reported more often by TGNC individuals and cis-women indicate low mood, emotional distress, trauma symptomology, body dissatisfaction, and suicidality contribute to significant gender differences in function endorsement. Differences between TGNC participants and cis-women were less pronounced, though TGNC individuals showed the greatest endorsement across most functions and versatility indicators. In all groups, social functions received far less endorsement than intrapersonal functions, indicating NSSI is primarily intra-personally motivated across gender groups. Specifically, affect regulation, tension reduction, and self-punishment were the most highly endorsed functions by each group. However, results also reveal important avenues for future gender-informed NSSI research, including investigation into reasons for generally low function endorsement by cis-men and the suitability of current NSSI assessments (developed from majority female samples) for capturing experiences of NSSI by people of all genders. Moreover, research is needed on the causes of the gender differences reported here, including societal inequalities such as gender-based violence and transphobic prejudice which feature prominently in models of gendered mental health disparities (Diamond, 2013; Hyde and Mezulis, 2020).

## Figures and Tables

**Table 1:** Comparison of NSSI function endorsement by category and domain between transgender and other gender non-conforming (TGNC) participants, cisgender women (Cis–W), and cisgender men (Cis-M) with histories of NSSI. The first 3 columns show the percentage of participants who endorse at least one item within each category/domain as often or always a reason for NSSI. Binary logistic regressions compared endorsement across gender groups. Bold type indicates significance at *p* < 0.05.

	TGNC %	Cis-W %	Cis-M %	Pairwise comparison	OR [95%CI]	*p*-Value
Intrapersonal functions	92.50	86.55	76.70	M vs W	1.84 [1.12, 3.00]	**0.015** ^a^
				M vs TGNC	3.32 [1.34, 8.26]	**0.010**
				W vs TGNC	1.81 [0.73, 4.45]	0.198
Affect regulation	88.75	83.79	68.75	M vs W	2.26 [1.44, 3.55]	**<** **0.001** ^a^
				M vs TGNC	3.33 [1.54, 7.20]	**0.002**
				W vs TGNC	1.47 [0.69, 3.16]	0.32
Self-punishment	71.79	53.41	48.55	M vs W	1.10 [0.76, 1.61]	0.61^a^
				M vs TGNC	2.36 [1.34, 4.16]	**0.003**
				W vs TGNC	2.13 [1.25, 3.64]	**0.005**
Anti-dissociation	50.00	42.50	22.54	M vs W	2.31 [1.50, 3.55]	**<** **0.001** ^a^
				M vs TGNC	2.99 [1.69, 5.30]	**<** **0.001**
				W vs TGNC	1.30 [0.79, 2.14]	0.31
Anti-suicide	31.25	24.14	13.64	M vs W	1.91 [1.15, 3.19]	**0.013** ^a^
				M vs TGNC	2.63 [1.38, 5.01]	**0.003**
				W vs TGNC	1.37 [0.80, 2.37]	0.26
Sensation seeking	25.00	27.59	17.05	M vs W	1.75 [1.09, 2.82]	**0.020** ^a^
				M vs TGNC	1.46 [0.77, 2.80]	0.25
				W vs TGNC	0.83 [0.47, 1.48]	0.54
Sexuality	1.25	2.76	3.41	M vs W	0.80 [0.27, 2.36]	0.69
				M vs TGNC	0.36 [0.04, 3.03]	0.35
				W vs TGNC	0.45 [0.05, 3.62]	0.45
Social functions	46.25	31.38	29.55	M vs W	1.09 [0.73, 1.64]	0.68
				M vs TGNC	2.05 [1.19, 3.54]	**0.010**
				W vs TGNC	1.88 [1.14, 3.12]	**0.014**
Interpersonal influence	36.25	24.83	28.41	M vs W	0.83 [0.55, 1.27]	0.39
				M vs TGNC	1.43 [0.82, 2.51]	0.21
				W vs TGNC	1.72 [1.02, 2.92]	**0.044**
Body image	19.74	13.77	2.92	M vs W	5.76 [1.83, 12.39]	**0.001** ^a^
				M vs TGNC	6.87 [2.38, 19.79]	**<0.001**
				W vs TGNC	1.44 [0.74, 2.80]	0.28

a Gender group comparisons included age as a covariate due to age being significantly associated with endorsement of this domain or category.

**Table 2:** Item-level comparison of NSSI functions between transgender and other gender non-conforming (TGNC) participants, cisgender women (Cis–W), and cisgender men (Cis-M). First three columns present the percentage of participants who endorsed each function as often or always a reason for NSSI. Binary logistic regressions compared endorsement across gender groups. Bold type indicates significance at *p* < 0.05.

	TGNC %	Cis-W %	Cis-M %	Pairwise comparison	OR [95%CI]	*p*-value
Intrapersonal functions						
Affect regulation						
Release unbearable tension	75.64	63.93	46.51	M vs W	2.04 [1.38, 3.00]	**<** **0.001**
				M vs TGNC	3.57 [1.96, 6.49]	**<** **0.001**
				W vs TGNC	1.75 [0.99, 3.10]	0.054
Release anger	51.95	52.50	43.68	M vs W	1.35 [0.92, 1.98]	0.13^b^
				M vs TGNC	1.25 [0.72, 2.16]	0.43
				W vs TGNC	0.93 [0.56, 1.55]	0.78
Release frustration	53.25	51.61	41.86	M vs W	1.48 [1.01, 2.17]	**0.044**
				M vs TGNC	1.58 [0.92, 2.72]	0.10
				W vs TGNC	1.07 [0.64, 1.77]	0.80
Relieve feelings of sadness or feeling “down”	53.85	55.48	30.81	M vs W	2.80 [1.88, 4.17]	**<0.001**
				M vs TGNC	2.62 [1.51, 4.54]	**0.001**
				W vs TGNC	0.94 [0.57, 1.55]	0.80
Stop feeling alone and empty	53.25	45.91	30.99	M vs W	1.82 [1.21, 2.72]	**0.004** ^b^
				M vs TGNC	2.36 [1.35, 4.13]	**0.003**
				W vs TGNC	1.30 [0.78, 2.16]	0.31
Experience physical pain in one area, when the other pain I feel is unbearable	57.14	46.43	19.65	M vs W	3.39 [2.17, 5.30]	**<0.001** ^b^
				M vs TGNC	5.00 [2.77, 9.04]	**<0.001**
				W vs TGNC	1.47 [0.88, 2.46]	0.14
To distract me from unpleasant memories	55.26	39.07	23.39	M vs W	2.01 [1.31, 3.10]	**0.001** ^b^
				M vs TGNC	3.75 [2.10, 6.70]	**<0.001**
				W vs TGNC	1.86 [1.11, 3.12]	**0.018**
Self-punishment						
Punish myself	71.79	53.41	48.55	M vs W	1.16 [0.79, 1.70]	0.45^b^
				M vs TGNC	2.49 [1.39, 4.45]	**0.002**
				W vs TGNC	2.14 [1.24, 3.71]	**0.006**
Anti-dissociation						
Produce a sense of being real when I feel numb and “unreal”	50.00	42.50	22.54	M vs W	2.40 [1.56, 3.70]	**<0.001** ^b^
				M vs TGNC	3.08 [1.73, 5.47]	**<0.001**
				W vs TGNC	1.28 [0.77, 2.13]	0.34
Anti-suicide						
Stop me from thinking about ideas of killing myself	28.95	22.46	12.28	M vs W	1.98 [1.15, 3.39]	**0.014** ^b^
				M vs TGNC	2.68 [1.36, 5.30]	**0.005**
				W vs TGNC	1.36 [0.77, 2.41]	0.30
Stop me from acting out ideas of killing myself	27.63	19.27	11.70	M vs W	1.80 [1.04, 3.14]	**0.037**
				M vs TGNC	2.88 [1.45, 5.72]	**0.002**
				W vs TGNC	1.60 [0.89, 2.87]	0.12
Sensation seeking						
Experience a “high” that feels like a drug high	15.79	16.91	6.43	M vs W	2.77 [1.39, 5.53]	**0.004** ^b^
				M vs TGNC	2.42 [1.08, 5.82]	**0.048**
				W vs TGNC	0.88 [0.44, 1.75]	0.71
Provide a sense of excitement that feels exhilarating	9.21	11.27	11.70	M vs W	0.96 [0.53, 1.74]	0.89
				M vs TGNC	0.77 [0.31, 1.90]	0.56
				W vs TGNC	0.80 [0.34, 1.89]	0.61
Prove to myself how much I can take	13.16	15.22	7.06	M vs W	2.19 [1.11, 4.32]	**0.023** ^b^
				M vs TGNC	1.76 [0.72, 4.30]	0.22
				W vs TGNC	0.80 [0.38, 1.69]	0.56
Sexuality						
For sexual excitement	0.00	2.20	1.75	M vs W	1.26 [0.31, 5.10]	0.75
				M vs TGNC	a	
				W vs TGNC	a	
To diminish feelings of sexual arousal	1.25	1.10	2.92	M vs W	0.37 [0.09, 1.56]	0.18
				M vs TGNC	0.44 [0.05, 3.85]	0.46
				W vs TGNC	1.20 [0.12,11.70]	0.88
Social Functions						
Interpersonal influence						
Get care or attention from other people	22.08	14.23	15.20	M vs W	0.93 [0.54, 1.58]	0.78
				M vs TGNC	1.58 [0.80, 3.12]	0.19
				W vs TGNC	1.71 [0.90, 3.23]	0.10
Show others how hurt or damaged I am	10.39	10.22	9.83	M vs W	0.96 [0.51, 1.83]	0.91^b^
				M vs TGNC	0.92 [0.38, 2.27]	0.86
				W vs TGNC	0.95 [0.42, 2.21]	0.92
Stop people from expecting so much from me	7.89	7.33	4.09	M vs W	1.85 [0.77, 4.48]	0.17
				M vs TGNC	2.01 [0.65, 6.19]	0.23
				W vs TGNC	1.08 [0.42, 2.80]	0.87
Stop my parents being angry with me	9.21	6.50	5.26	M vs W	1.25 [0.55, 2.85]	0.59
				M vs TGNC	1.83 [0.65, 5.10]	0.25
				W vs TGNC	1.46 [0.59, 3.64]	0.42
Belong to a group	0.00	3.30	3.51	M vs W	0.94 [0.33, 2.68]	0.90
				M vs TGNC	a	
				W vs TGNC	a	
Get out of doing something I don't want to do	2.63	2.93	4.09	M vs W	0.71 [0.25, 1.99]	0.51
				M vs TGNC	0.63 [0.13, 3.12]	0.58
				W vs TGNC	0.89 [0.19, 4.31]	0.89
Avoid getting into trouble for something I did	0.00	3.28	4.09	M vs W	0.80 [0.29, 2.18]	0.66
				M vs TGNC	a	
				W vs TGNC	a	
Body image						
Change my body image and/or appearance	19.74	13.77	2.92	M vs W	4.87 [1.87, 12.70]	**0.001** ^b^
				M vs TGNC	7.03 [2.43, 20.32]	**<0.001**
				W vs TGNC	1.44 [0.74, 2.81]	0.28

a Unable to test comparisons due to zero endorsement by TGNC participants.

b Gender group comparisons included age as a covariate due to age being significantly associated with endorsement of this item.

**Table 3:** Comparisons of NSSI function versatility between transgender and other gender non-conforming (TGNC) participants, cisgender women (Cis–W), and cisgender men (Cis-M). Gender groups were compared with linear regressions including age as a covariate. Bold type indicates significance at *p* < 0.05.

	TGNC M(SD)	Cis-W M(SD)	Cis-M M(SD)	Pairwise comparison	Coef. [95%CI]	*Cohen's d*	*p*-value
Total number of domains endorsed (max. 8)	3.2 (1.72)	2.69 (1.78)	2.04 (1.63)	M vs W	1.63 [0.91, 2.34]	−0.38	**<0.001**
				M vs TGNC	2.40 [1.39, 3.40]	-0.69	**<0.001**
				W vs TGNC	0.76 [−0.20,1.73]	−0.29	0.12
Total number of items endorsed (max. 19)	6.65 (3.81)	5.78 (4.19)	4.02 (3.65)	M vs W	0.58 [0.27, 0.90]	−0.45	**<0.001**
				M vs TGNC	1.04 [0.59, 1.50]	−0.70	**<0.001**
				W vs TGNC	0.46 [0.03, 0.89]	−0.22	**0.035**

**Table 4:** Proportions of transgender and other gender non-conforming (TGNC) participants, cisgender women (Cis–W), and cisgender men (Cis-M) who endorsed intrapersonal and/or social functions as often or always a reason for NSSI. Gender groups were compared with binary logistic regressions including age as a covariate. Bold type indicates significance at *p* < 0.05.

	TGNC %	Cis- W %	Cis-M %	Pairwise comparison	OR [95% CI]	*p*-value
Intrapersonal functions only	47.50	57.59	51.14	M vs W M vs TGNC W vs TGNC	1.30 [0.89, 1.89] 0.86 [0.51, 1.47] 0.67 [0.41, 1.10]	0.18 0.59 0.11
Both intrapersonal and social functions	45.00	28.97	25.57	M vs W M vs TGNC W vs TGNC	1.19 [0.78, 1.81] 2.38 [1.37, 4.15] 2.01 [1.21, 3.34]	0.43 **0.002** **0.007**
Social functions only	1.25	2.41	3.98	M vs W M vs TGNC W vs TGNC	0.60 [0.21, 1.73] 0.31 [0.04, 2.53] 0.51 [0.06, 4.22]	0.34 0.27 0.53
Neither intrapersonal nor social functions	6.25	11.03	19.31	M vs W M vs TGNC W vs TGNC	0.52 [0.31, 0.88] 0.28 [0.10, 0.74] 0.54 [0.20, 1.43]	**0.014** **0.011** 0.21

## Data Availability

Requests for de-identified data can be made to the Chief Investigator Prof Samuel R. Chamberlain for consideration.

## Abbreviations

NSSI: Non-suicidal self-injury
TGNC: Transgender and other gender non-conforming
Cis: Cisgender
OSI-F: Ottawa Self-Injury Inventory Functions Scale
